# Induced sputum versus bronchoalveolar lavage in lower respiratory tract infection: an exploratory paired diagnostic study

**DOI:** 10.3389/fmed.2026.1845078

**Published:** 2026-08-31

**Authors:** Jiaxi Deng, Haimao Qin, Jie Yang, Yuanyuan Li, Dengfeng Zhou, Bo Zhang, Yushu Ruan

**Affiliations:** Department of Pulmonary and Critical Care Medicine, Wuhan Fourth Hospital, Wuhan, Hubei, China

**Keywords:** bronchoalveolar lavage fluid, clinical adjudication, exploratory study, induced sputum, lower respiratory tract infection, targeted next-generation sequencing

## Abstract

**Introduction:**

Bronchoalveolar lavage fluid (BALF) is widely used for etiologic assessment of lower respiratory tract infection (LRTI), but its invasiveness limits routine use. This study evaluated induced sputum (IS) as a less-invasive specimen for targeted next-generation sequencing (tNGS).

**Methods:**

In this prospective exploratory study, paired IS and BALF samples from 30 adults with suspected LRTI underwent tNGS. The primary etiology was determined by composite clinical adjudication. Detected microorganisms were classified as definitive pathogens, conditional pathogens, or colonizers/commensals.

**Results:**

Clinical-match positivity was 70.0% (21/30) for IS and 86.7% (26/30) for BALF, while combining both specimens increased coverage to 93.3% (28/30). Definitive pathogen detection was broadly similar between specimens, whereas colonizers/commensals were more frequently detected in IS (45.6% vs 28.2%). After excluding six *Mycoplasma*/*Mycobacterium* cases, clinical-match positivity was 75.0% for IS and 83.3% for BALF (McNemar exact *p* = 0.688).

**Discussion:**

Differences between IS and BALF tNGS were mainly attributable to upper-airway background signals. IS may serve as a less-invasive initial option in selected non-complex LRTIs, though further prospective validation in larger cohorts is needed.

## Background

Lower respiratory tract infections (LRTIs) are among the most common and deadliest infectious diseases worldwide, posing a substantial health threat across all age groups (1). This burden is particularly pronounced in vulnerable populations with chronic comorbidities or impaired immunity, in whom progression to severe pneumonia may be associated with case-fatality rates as high as 50% (2), thereby imposing considerable healthcare utilization and socioeconomic costs (3). Clinical evidence indicates that inappropriate or delayed initial antimicrobial therapy is a major contributor to clinical deterioration (2). Accordingly, timely and accurate etiological diagnosis has become pivotal for improving outcomes and for curbing the emergence and spread of antimicrobial resistance. However, widely used conventional diagnostic approaches—including culture, serologic testing, and single-target PCR—are often limited by low sensitivity and long turnaround times, and they may fail to reliably identify fastidious organisms or mixed infections (4). To overcome these limitations, multiplex syndromic PCR panels have been widely adopted in clinical practice. These panels offer a rapid, highly sensitive, and cost-effective approach to simultaneously screen for multiple common respiratory pathogens within a single assay (5). However, the pathogen targets on syndromic panels are pre-specified and fixed, meaning they remain blind to rare, emerging, or highly polymorphic microorganisms, and their capacity to delineate complex polymicrobial profiles or comprehensive resistance markers can be restricted (4, 6). Consequently, large cohort studies have shown that even with comprehensive conventional workups and routine molecular testing, the causative pathogen remains unidentified in up to 62% of community-acquired pneumonia (CAP) cases (5). To address this remaining diagnostic gap, next-generation sequencing (NGS) can meaningfully increase etiologic detection rates (6). Although metagenomic NGS (mNGS) offers broad, hypothesis-free detection, its high cost and interpretive complexity often limit routine implementation. By contrast, targeted next-generation sequencing (tNGS), particularly multiplex PCR-based tNGS that uses predefined pathogen-enrichment targets followed by a sequencing-based readout, has demonstrated comparable clinical utility in guiding antimicrobial optimization and improving patient outcomes in many settings, while meeting the practical diagnostic needs of most patients with LRTIs (7, 8).

NGS using bronchoalveolar lavage fluid (BALF) is widely regarded as a reference approach for etiologic assessment of lower respiratory tract infections (9), yet its invasive nature limits routine use in patients with mild-to-moderate disease or in those who cannot tolerate bronchoscopy. In contrast, spontaneously expectorated sputum is prone to contamination by oropharyngeal colonizing flora, which can increase background noise and complicate interpretation of NGS results (10). Induced sputum (IS), obtained via nebulized hypertonic saline inhalation (11), offers a less-invasive sampling option and may reduce upper-airway contamination while improving recovery of lower-airway pathogens. Therefore, using a multiplex targeted amplification-based tNGS, this exploratory paired diagnostic study compared IS and BALF specimens from the same patients with suspected LRTI to evaluate whether IS can provide clinically informative pathogen detection as a less-invasive sampling option.

## Materials and methods

### Patients and study design

This prospective exploratory study enrolled consecutive adult patients with suspected LRTI admitted to Wuhan Fourth Hospital between January 2026 and March 2026. Patient recruitment was initiated concurrently across the three campuses of Wuhan Fourth Hospital, which together provide 180 inpatient beds. Importantly, eligibility was based on clinical and radiologic criteria for suspected LRTI rather than on a pre-specified pathogen, viral syndrome, or outbreak-related screening strategy. Therefore, the cohort was intended to represent a clinically suspected LRTI population requiring etiologic evaluation, rather than a single pathogen-driven or virus-enriched population. Pediatric patients, long-term residential care facility residents, known immunocompromised patients, and patients receiving invasive mechanical ventilation were not represented in this cohort. The study was approved by the institutional review board of Wuhan Fourth Hospital (approval KY2026-014-01), and was conducted in accordance with the Declaration of Helsinki and its subsequent amendments. Written informed consent was obtained from all participants (or their legal representatives), and all patient data were anonymized prior to analysis.

Suspected LRTI was defined by the presence of (i) new or progressive pulmonary infiltrates, consolidation, or ground-glass opacities on chest imaging, and (ii) at least one of the following clinical criteria: (1) body temperature > 37.0°C; (2) at least one respiratory symptom including cough, sputum production, dyspnea, chest pain, or abnormal breath sounds on auscultation; or (3) peripheral blood leukocyte count > 10 × 10^9^/L or < 4 × 10^9^/L.

Patients were excluded if (i) IS and/or BALF could not be obtained (e.g., refusal or contraindication); (ii) systemic antibiotic therapy within 14 days prior to the current admission/sample collection; (iii) either specimen failed laboratory quality control for tNGS (defined as a total sequence output < 20 million reads, a Q30 ratio < 85%, or internal process control failure as specified by the testing laboratory); (iv) paired testing was not possible due to missing specimens; or (v) clinical data were insufficient for final adjudication (defined as the lack of complete baseline chest computed tomography CT scans, essential dynamic inflammatory biomarker records, or post-treatment follow-up data required to establish the composite clinical reference standard). No patients were actually excluded under criterion (vi) in this cohort, as all enrolled patients had complete core clinical data. The precise criteria for downstream clinical adjudication are detailed in Supplementary Table S1.

### Specimen collection

Induced sputum was collected using a standardized hypertonic saline inhalation protocol. Patients were instructed to rinse the mouth prior to induction, and expectorated sputum was collected into sterile containers, preferentially selecting mucoid plugs and avoiding saliva contamination. BALF was obtained by bronchoscopy according to routine clinical procedures; lavage was performed in the segment corresponding to radiographic abnormalities whenever feasible. Upon admission, serum specimens and spontaneously expectorated sputum were also collected from all patients for routine culture and serologic testing. All specimens were transported to the microbiology laboratory promptly and processed according to standard operating procedures.

### Conventional microbiological tests

For conventional microbiological tests (CMTs), routine culture-based testing was performed according to the standard operating procedures of the hospital microbiology laboratory. The CMT workup included routine bacterial and fungal cultures of spontaneously expectorated sputum and BALF specimens. Routine bacterial culture was intended to recover common aerobic and facultative bacterial respiratory pathogens, whereas fungal culture was intended to recover clinically relevant yeasts and molds according to routine laboratory practice. Briefly, respiratory specimens were inoculated onto blood agar, chocolate agar, and MacConkey agar for routine bacterial culture and onto Sabouraud dextrose agar for fungal culture, followed by incubation under standard laboratory conditions.

In addition, a multiplex respiratory pathogen nucleic acid panel (six targets: influenza A virus, influenza B virus, respiratory syncytial virus, adenovirus, *Mycoplasma pneumoniae*, and *Chlamydia pneumoniae*) was performed on sputum specimens to screen for common viral and atypical respiratory pathogens. A nine-target respiratory pathogen IgM antibody panel (including *Legionella pneumophila*, *Mycoplasma pneumoniae*, *Chlamydia pneumoniae*, adenovirus, respiratory syncytial virus, influenza A virus, influenza B virus, parainfluenza virus, and Coxiella burnetii) was also performed on serum specimens. For mycobacterial infection, acid-fast bacilli (AFB) staining and Xpert MTB/RIF were performed on clinically indicated BALF specimens, and mycobacterial culture was performed when clinically warranted. Viral culture, anaerobic culture, and urinary antigen testing were not performed as part of the routine CMT workup in this study. The CMT results were used as a descriptive real-world comparator and as one component of the composite clinical adjudication, but they were not treated as a complete microbiological reference standard. Microorganisms not recoverable by conventional culture were excluded from culture-based sensitivity calculations to ensure a fair comparison with tNGS.

### Targeted next-generation sequencing

#### NGS assay

A multiplex targeted amplification–based tNGS assay was performed for pathogen detection in paired IS and BALF specimens. Testing was conducted in the Wuhan KingMed laboratory using a standardized workflow and commercially available reagents and instruments.

#### Sample processing and nucleic acid extraction

Respiratory specimens were processed according to the laboratory standard operating procedure to obtain a homogeneous suspension suitable for downstream molecular testing. Total nucleic acids were extracted using a pathogen DNA/RNA extraction kit (MagPure; Cat. R6672B-F) on an automated extraction platform (Auto-Pure96).

#### Library preparation and sequencing

Target enrichment and library construction were performed using a dedicated respiratory tNGS library preparation kit (KingCreate; Cat. KS608-100HXD96). PCR amplification was carried out on a Veriti thermal cycler. Library quantity and fragment profiles were assessed using Qubit 4.0 fluorometry and Qsep100 electrophoretic analysis, respectively. Sequencing was performed on the KM MiniSeqDx-CN platform (KingCreate) with a universal sequencing reagent kit (Cat. KS107-CXR).

#### Quality control and analytical sensitivity

The analytical limit of detection (LoD) was determined by an external testing laboratory (KingMed) using probit analysis of serial dilution panels. To validate the ∼225-target tNGS panel, analytical sensitivity was evaluated using a representative cohort of 17 pathogens selected across major taxonomic groups (fungi, bacteria, mycobacteria, DNA viruses, and RNA viruses). This selection effectively established the analytical performance boundaries of the assay; across these validated representatives, LoD values ranged from 50 copies/mL (*Mycobacterium tuberculosis* complex) to 2,000 copies/mL (Enterovirus A71), while LoDs for non-mycobacterial bacteria and fungi ranged from 250 to 1,000 CFU/mL. The complete representative LoD dataset is provided in Supplementary Table S2. Microorganisms present at concentrations below these established thresholds may not be reliably detected.

#### Bioinformatic analysis and reporting

Sequencing data were processed by the testing laboratory using a standardized bioinformatic pipeline to generate organism-level detection results based on reads/target signals against a curated reference scope for respiratory pathogens.

#### Interpretation

For result interpretation, amplicon coverage and normalized read counts were used as primary indicators. Detection thresholds were pre-specified: for bacteria, fungi, and uncommon respiratory pathogens, amplicon coverage ≥ 50% and normalized read count ≥ 10; for viruses, amplicon coverage ≥ 50% and normalized read count ≥ 3, or normalized read count ≥ 10; for Mycobacterium tuberculosis complex, normalized read count ≥ 1. Detected microorganisms meeting the above signal thresholds were indexed against a curated reference table of established respiratory pathogens adapted from the framework described by Langelier et al. (12). Organisms matching entries in this reference table were designated as putative pathogens. The complete list of target pathogens covered by the tNGS panel is provided in Supplementary Table S3.

#### Clinical adjudication and reference standard

Because no single microbiological assay can serve as a perfect gold standard for LRTI, the final etiologic diagnosis was determined using a composite clinical adjudication approach. Two experienced clinicians independently reviewed each case using pre-specified criteria, incorporating clinical presentation, radiologic findings, host factors, inflammatory laboratory parameters, conventional microbiological results, paired IS and BALF tNGS results, antimicrobial treatment, and clinical response. Disagreements were resolved by discussion, and, if necessary, by consultation with a third senior clinician.

Microbiological findings were classified as clinically concordant, clinically ambiguous, or clinically discordant according to the pre-specified criteria in Supplementary Table S1. Only microorganisms classified as clinically concordant with the adjudicated primary etiology were counted as clinical-match positive in the main analysis. Microorganisms classified as clinically ambiguous or clinically discordant were retained in descriptive microbial-spectrum analyses but were not counted as etiologic matches. It was consistent with prior studies using composite reference standards (13, 14).

Additionally, in accordance with the previous research (12), all tNGS detections were independently classified by two clinicians into three tiers based on pathogenic potential: Category A—definitive respiratory pathogens with well-established causal association; Category B—conditional pathogens capable of causing disease under specific host conditions; and Category C—typical colonizers/commensals of the upper respiratory or oropharyngeal microbiota.

Clinical-match positivity (CMP) was defined at the patient level as the proportion of patients in whom a given diagnostic method or specimen detected at least one microorganism adjudicated as clinically concordant with the final primary etiologic diagnosis. Microorganisms classified as clinically ambiguous or clinically discordant were not counted as clinical-match positive.

#### Statistical analysis

Quantitative variables were expressed as medians with ranges, and categorical variables as counts with percentages. The paired performance between IS and BALF was evaluated using concordance analyses, including overall agreement and positive percent agreement (PPA).

Patient-level positive percent agreement (PPA) was calculated as:


PPA=TP/(TP+FN)×100%

where TP (true positive): cases in which both IS tNGS and BALF tNGS were concordant with the adjudicated clinical etiology; FN (false negative): cases in which BALF tNGS was concordant with the adjudicated etiology but IS tNGS was not. BALF tNGS served as the reference standard for PPA calculation, consistent with the study objective of evaluating IS tNGS as a potential noninvasive surrogate for BALF.

Paired proportions were compared using McNemar’s test where applicable. A two-sided *p*-value < 0.05 was considered statistically significant. Statistical analyses were performed using SPSS 22.0 software (IBM, Armonk, NY, United States).

## Results

### Baseline characteristics

Between January 2026 and March 2026, 30 consecutive eligible patients with suspected LRTI were enrolled and included in the paired analysis. All patients underwent tNGS testing, and conventional microbiological cultures were performed using spontaneously expectorated sputum and BALF. In addition, paired induced sputum and BALF specimens were collected for tNGS. None of the patients had received antimicrobial therapy prior to sampling. Baseline demographics and clinical characteristics are summarized in Table 1. Among the 30 patients, 66.7% were male and the median age was 64 years. The most common symptoms were cough (63.3%), fever (26.7%), and dyspnea (16.7%), and 60.0% of patients had a history of smoking. Elevated leukocyte counts were observed in 13 patients (43.3%), and an increased neutrophil proportion was noted in 20 patients (66.7%). The median levels of inflammatory biomarkers levels of procalcitonin, C-reactive protein, and erythrocyte sedimentation ratein 20 patients (66.7ized in e collAmong underlying chronic pulmonary conditions, COPD was the most frequent (16.7%).

**TABLE 1:** Baseline characteristics of 30 patients.

Characteristic	Value [median (range) or no. (%)]
Age	
Years	64 (24, 91)
Distribution	
19∼40 Years	3 (10.0%)
41∼60 Years	11 (36.7%)
> 60 Years	16 (53.3%)
Male sex	20 (66.7%)
Onset symptoms	
Fever	8 (26.7%)
Dyspnea	5 (16.7%)
Cough	19 (63.3%)
Smoking history	
Never smoker	12 (40.0%)
Former/current smoker	18 (60.0%)
Inflammation biomarker	
WBC (10^9^/L)	9.83 (3.85, 19.25)
NEU (%)	76.17 (56.4, 91.2)
LYM (10^9^/L)	1.53 (0.31, 3.12)
PCT (ng/mL)	0.21 (0.04, 1.58)
CRP (mg/L)	88 (6, 189)
ESR (mm/h)	47 (10, 93)
Chronic pulmonary disease	
COPD	5 (16.7%)
Bronchiectasis	2 (6.7%)
Asthma	1 (3.3%)
Interstitial lung disease	1 (3.3%)
Lung cancer	3 (10.0%)
Comorbidity	
Diabetes mellitus	9 (30%)
Liver disease	4 (13.3%)
Cerebrovascular disease	5 (16.7%)
Kidney disease	5 (16.7%)
Severity score	
APACHE II	8 (3, 17)
SOFA	6 (3, 12)
Length of stay (days)	10 (5, 27)

WBC, white blood cell; NEU, neutrophil; LYM, lymphocyte; PCT, procalcitonin; CRP, C-reactive protein; ESR, erythrocyte sedimentation rate;. COPD, chronic obstructive pulmonary disease; APACHE II, acute physiology and chronic health evaluation II; SOFA, sequential organ failure assessment.

### Comparison of clinical-match positivity between paired IS and BALF tNGS

**FIGURE 1:**
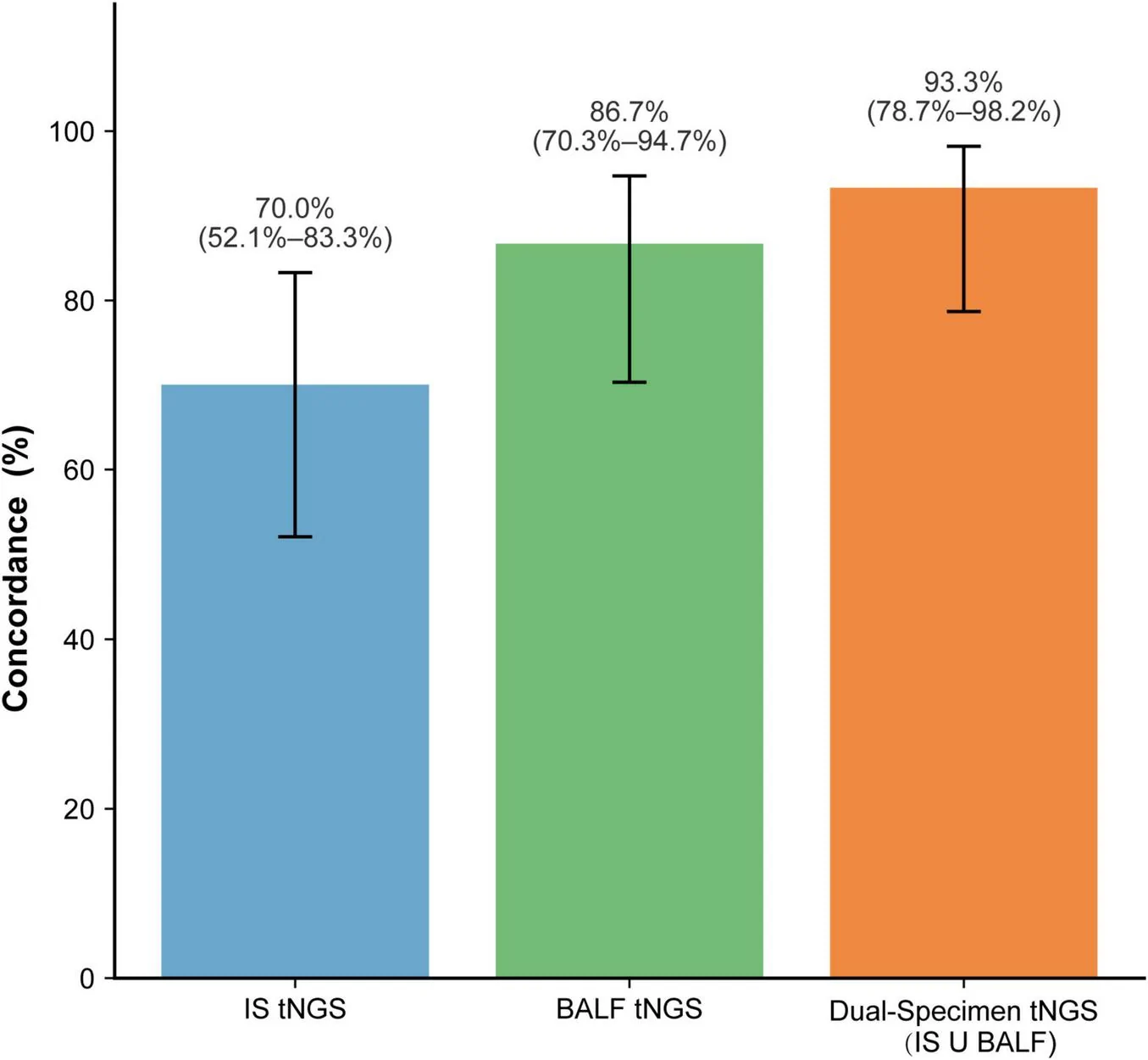
Concordance of IS, BALF, and dual-specimen tNGS with adjudicated etiology. Concordance was assessed against the adjudicated primary clinical etiology in 30 patients. IS tNGS, BALF tNGS, and the dual-specimen strategy (IS or BALF) showed concordance rates of 70.0% (21/30), 86.7% (26/30), and 93.3% (28/30), respectively. Error bars indicate 95% confidence intervals calculated using the Wilson score method. IS, induced sputum; BALF, bronchoalveolar lavage fluid; tNGS, targeted next-generation sequencing.

Using the adjudicated primary clinical etiology as the reference standard, we evaluated diagnostic performance in the 30 enrolled patients. As shown in Figure 1, by individual specimen, concordance was 70.0% (21/30; 95% CI 52.1–83.3%) for IS tNGS and 86.7% (26/30; 95% CI 70.3–94.7%) for BALF tNGS. The dual-specimen tNGS strategy (IS U BALF) achieved concordance of 93.3% (28/30; 95% CI 78.7–98.2%) with the adjudicated etiology.

### Evaluation of tNGS performance in different specimens

**FIGURE 2:**
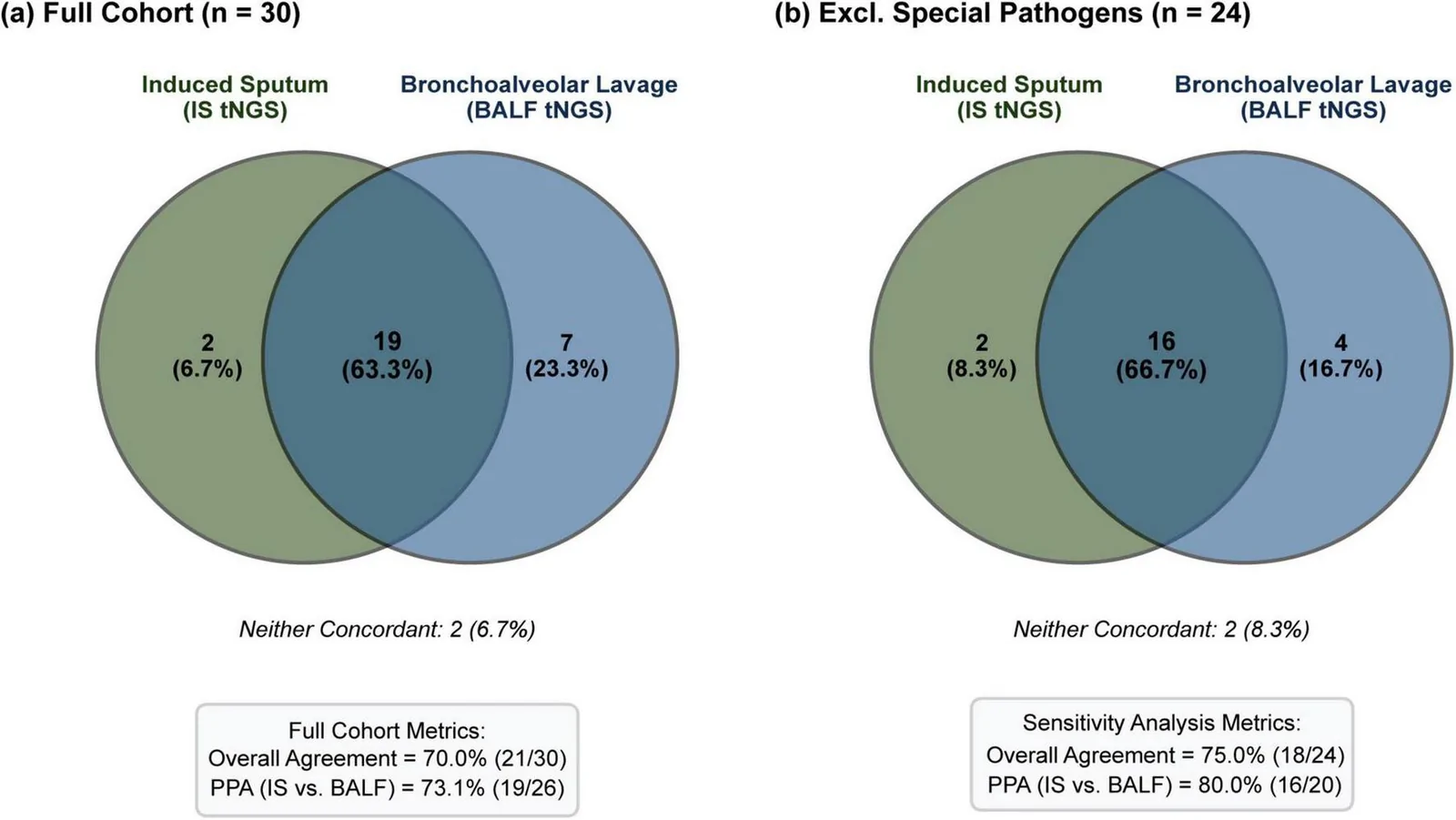
Paired concordance between IS and BALF tNGS with the adjudicated clinical etiology. **(a)** In the full cohort (*n* = 30), both specimens were concordant in 63.3% (19/30), BALF-only in 23.3% (7/30), IS-only in 6.7% (2/30), and neither in 6.7% (2/30), yielding an overall agreement of 70.0% (21/30) and a PPA of 73.1% (19/26). **(b)** After excluding cases adjudicated as *Mycoplasma* and *Mycobacterium* spp. (*n* = 24), overall agreement was 75.0% (18/24) and PPA was 80.0% (16/20). PPA was calculated with BALF tNGS as the reference.

In this cohort, tNGS based on a single specimen carried a non-negligible risk of missed detection or clinical discordance. By contrast, a dual-specimen strategy substantially increased clinical etiologic coverage: when “either induced sputum or BALF tNGS concordant with the clinical diagnosis” was used as the criterion, the overall clinical identification coverage reached 93.3% (28/30; 95% CI 78.7–98.2%), exceeding that of either specimen alone (Figure 1). Detailed paired analysis (Figure 2 and Table 2) showed that 63.3% of cases (19/30; 95% CI 45.5–78.1%) were clinically matched by tNGS in both induced sputum and BALF. BALF-only clinical matching occurred in 23.3% (7/30; 95% CI 11.8–40.9%), induced sputumching occurred in 6.7% (2/30; 95% CI 1.8–21.3%), and neither specimen matched the clinical diagnosis in only 6.7% (2/30; 95% CI 1.8–21.3%).

**TABLE 2:** Paired concordance between IS and BALF tNGS.

IS tNGS	BALF match +	BALF match -	Total
IS match +	19	2	21
IS match -	7	2	9
Total	26	4	30

Following the specimen-level performance comparison, we further explored whether the observed discordance between induced sputum (IS) and BALF was attributable to specific etiologic contexts rather than reflecting a global limitation of IS sampling. We therefore conducted a prespecified sensitivity analysis excluding cases adjudicated as predefined atypical/mycobacterial pathogens—defined a priori as Mycoplasma and Mycobacterium spp. (including TB/NTM). As shown in Figure 2 and Table 3, after exclusion (6 cases), IS concordance was 75.0% (18/24; 95% CI 55.1–88.0%) and BALF concordance was 83.3% (20/24; 95% CI 64.1–93.3%), with an overall agreement of 75.0% (18/24; 95% CI 55.1–88.0%) and a PPA of 80.0% (16/20; 95% CI 58.4–91.9%), calculated with BALF tNGS as the reference. The performance gap between IS and BALF narrowed considerably compared with the overall cohort, suggesting that the incremental diagnostic yield of BALF over IS is largely concentrated in special-pathogen scenarios. Notably, this analysis was performed to assess robustness and should be interpreted alongside the overall-cohort results.

**TABLE 3:** Paired concordance between IS and BALF tNGS after excluding predefined atypical/mycobacterial pathogens.

IS tNGS	BALF match +	BALF match -	Total
IS match +	16	2	18
IS match -	4	2	6
Total	20	4	24

Positivity indicates clinical-match positivity based on clinical adjudication. IS positivity was 70.0% (21/30) and BALF positivity was 86.7% (26/30); union coverage (IS ∪ BALF) was 93.3% (28/30).

Positivity indicates clinical-match positivity based on clinical adjudication after excluding predefined atypical/mycobacterial pathogens (*Mycoplasma* and *Mycobacterium* spp., *n* = 6). IS positivity was 75.0% (18/24) and BALF positivity was 83.3% (20/24); union coverage (IS ∪ BALF) was 91.7% (22/24). Overall agreement was 75.0% (18/24) and positive percent agreement (PPA) was 80.0% (16/20), calculated with BALF tNGS as the reference standard. McNemar exact test *p* = 0.688.

### tNGS microbial spectrum in IS and BALF

**FIGURE 3:**
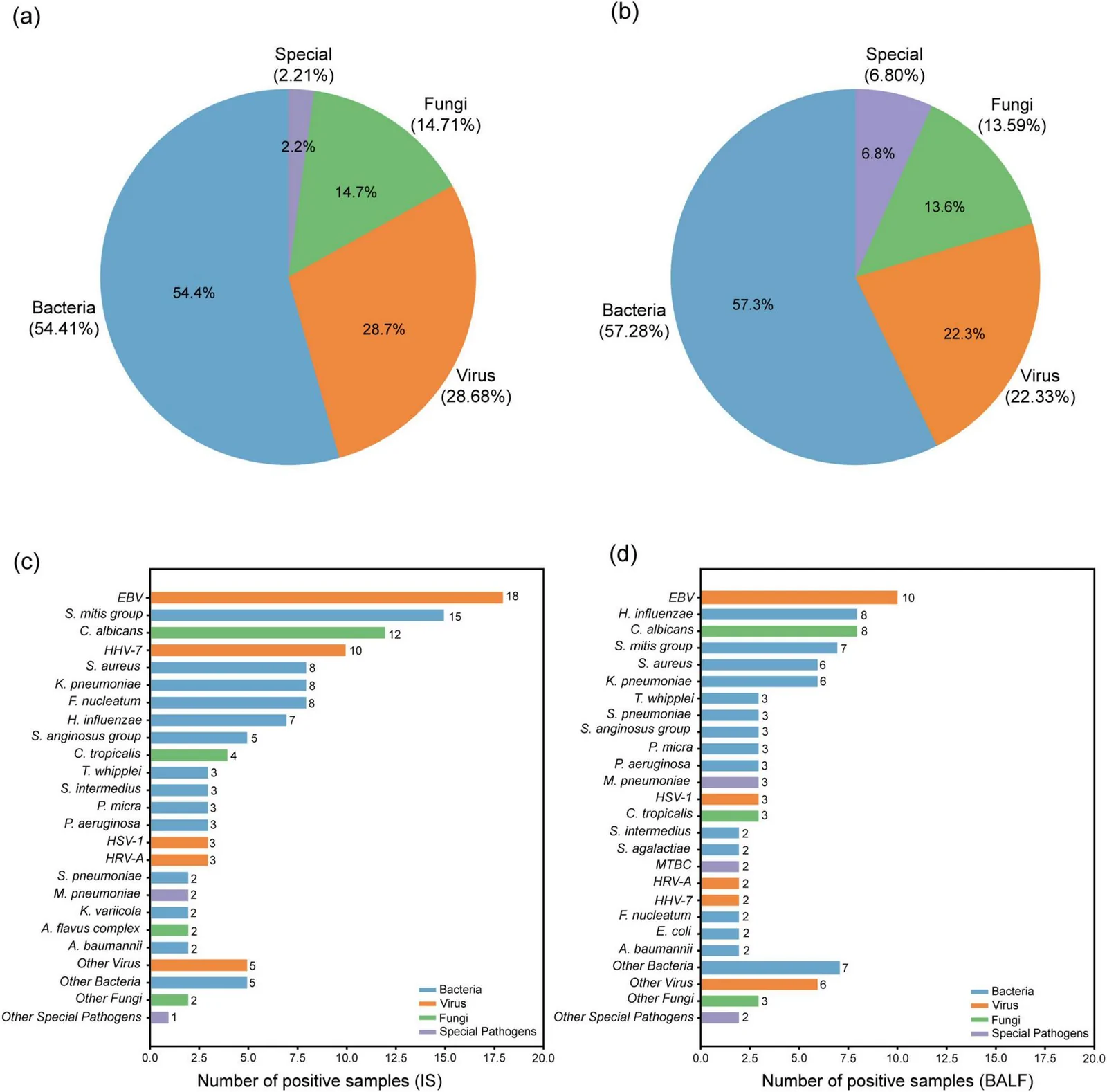
Microbial spectrum detected by tNGS in IS and BALF workflows. **(a,b)** Distribution of microorganisms detected by IS and BALF assays by categories, respectively. **(c,d)** Top detected microorganisms in IS and BALF, respectively. Individual taxa with at least two detections are shown separately, while the remaining low-frequency detections are grouped as “Other Bacteria,” “Other Virus,” “Other Fungi,” and “Other Special Pathogens.” Bar length represents the number of detections.

IS workflow identified a total of 136 microorganisms (Figure 3), dominated by bacteria and viruses. Bacteria comprised 54.41% (74/136) of all detections and were distributed across 18 species. The most frequently detected bacterial taxa were the *Streptococcus mitis* group (15/74, 20.27%), *Staphylococcus aureus* (8/74, 10.81%), *Klebsiella pneumoniae* (8/74, 10.81%), and *Fusobacterium nucleatum* (8/74, 10.81%). Viruses accounted for 28.68% (39/136) of detections across nine species, with Epstein–Barr virus (EBV; 18/39, 46.15%) and human betaherpesvirus 7 (HHV-7; 10/39, 25.64%) being the most prevalent. Fungi were detected in five species [20 isolates, 14.71% (20/136)], predominantly *Candida albicans* (12/20, 60.00%). In addition, special pathogens were detected in three samples (2.21% of all detections), including *Mycoplasma pneumoniae* (2/3, 66.67%) and *Mycobacterium tuberculosis* complex (1/3, 33.33%).

In comparison, the BALF workflow identified 103 microorganisms. Bacteria comprised 57.28% (59/103) of detections across 21 species. Although the overall bacterial proportion was similar to that in IS, the distribution differed: *Haemophilus influenzae* was most frequently detected (8/59, 13.56%), followed by the *Streptococcus mitis* group (7/59, 11.86%), and *Staphylococcus aureus* and *Klebsiella pneumoniae* (each 6/59, 10.17%). Viruses represented 22.33% (23/103) of detections across 10 species, with EBV remaining the most common (10/23, 43.48%). Fungi were detected in five species (14/103, 13.59%), again mainly *Candida albicans* (8/14, 57.14%). Notably, BALF yielded seven special-pathogen detections (6.80%), including *Mycoplasma pneumoniae* (3/7, 42.86%), *Mycobacterium tuberculosis* complex (2/7, 28.57%), *Mycobacterium kansasii* (1/7, 14.29%), and *Mycobacterium abscessus* subsp. *abscessus* (1/7, 14.29%), suggesting a potential advantage of BALF for identifying atypical pathogens.

We further stratified all tNGS detections by pathogenic potential (Supplementary Table S4). In IS, 19.1% (26/136) of detections were Category A definitive pathogens, 35.3% (48/136) Category B, and 45.6% (62/136) Category C colonizers. In BALF, the corresponding proportions were 29.1% (30/103), 42.7% (44/103), and 28.2% (29/103). Notably, the excess detections in IS were predominantly concentrated in Category C (62 vs. 29; Δ +17.4 pp), whereas Category A detections were comparable between specimens.

**FIGURE 4:**
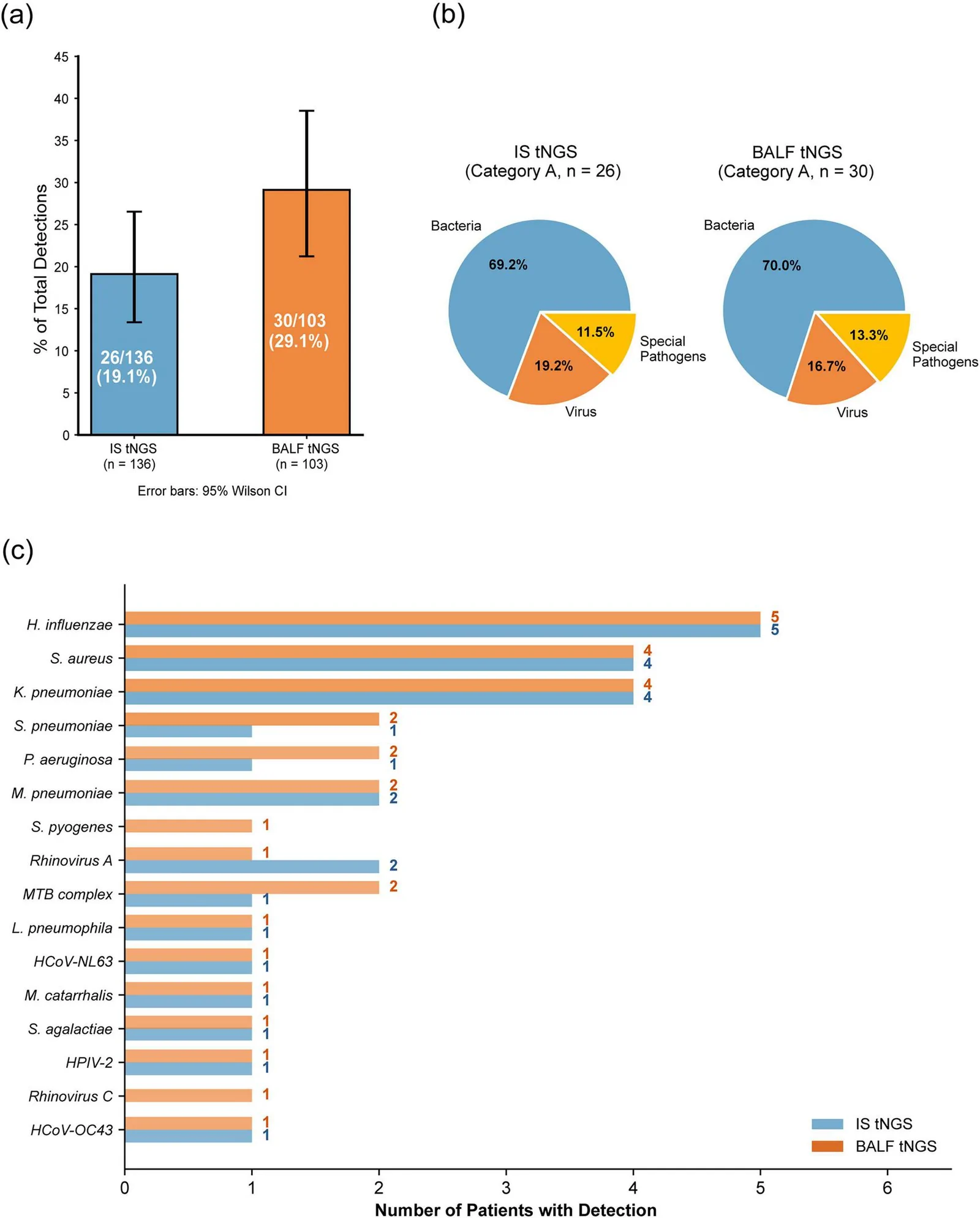
Composition and paired detection of category A definitive pathogens in IS and BALF tNGS. **(a)** Proportion of Category A detections among total tNGS detections in IS and BALF, respectively. **(b)** Taxonomic composition of Category A detections in IS and BALF, respectively. Bacteria, virus, and special-pathogen components are shown with corresponding percentages. **(c)** Paired detection counts of 16 Category A organisms in IS and BALF. Light blue bars indicate IS detections; orange bars indicate BALF detections. Numbers beside bars represent the number of patients with detection.

To further characterize the definitive pathogens, we examined the taxonomic composition and paired specimen distribution of Category A organisms (Figure 4). In both IS and BALF, Category A detections were predominantly bacterial (IS: 69.2%; BALF: 70.0%), with smaller contributions from viruses (19.2% vs. 16.7%) and special pathogens (11.5% vs. 13.3%). At the individual-organism level, detection counts were identical or nearly identical between IS and BALF for most definitive pathogens—including H. influenzae (5 vs. 5), *S. aureus* (4 vs. 4), and *K. pneumoniae* (4 vs. 4)—while the few between-specimen differences were limited to a small number of targets, most notably MTB complex (1 in IS vs. 2 in BALF) and Rhinovirus A (2 vs. 1).

### Concordance and quantitative comparison of specific pathogens

**FIGURE 5:**
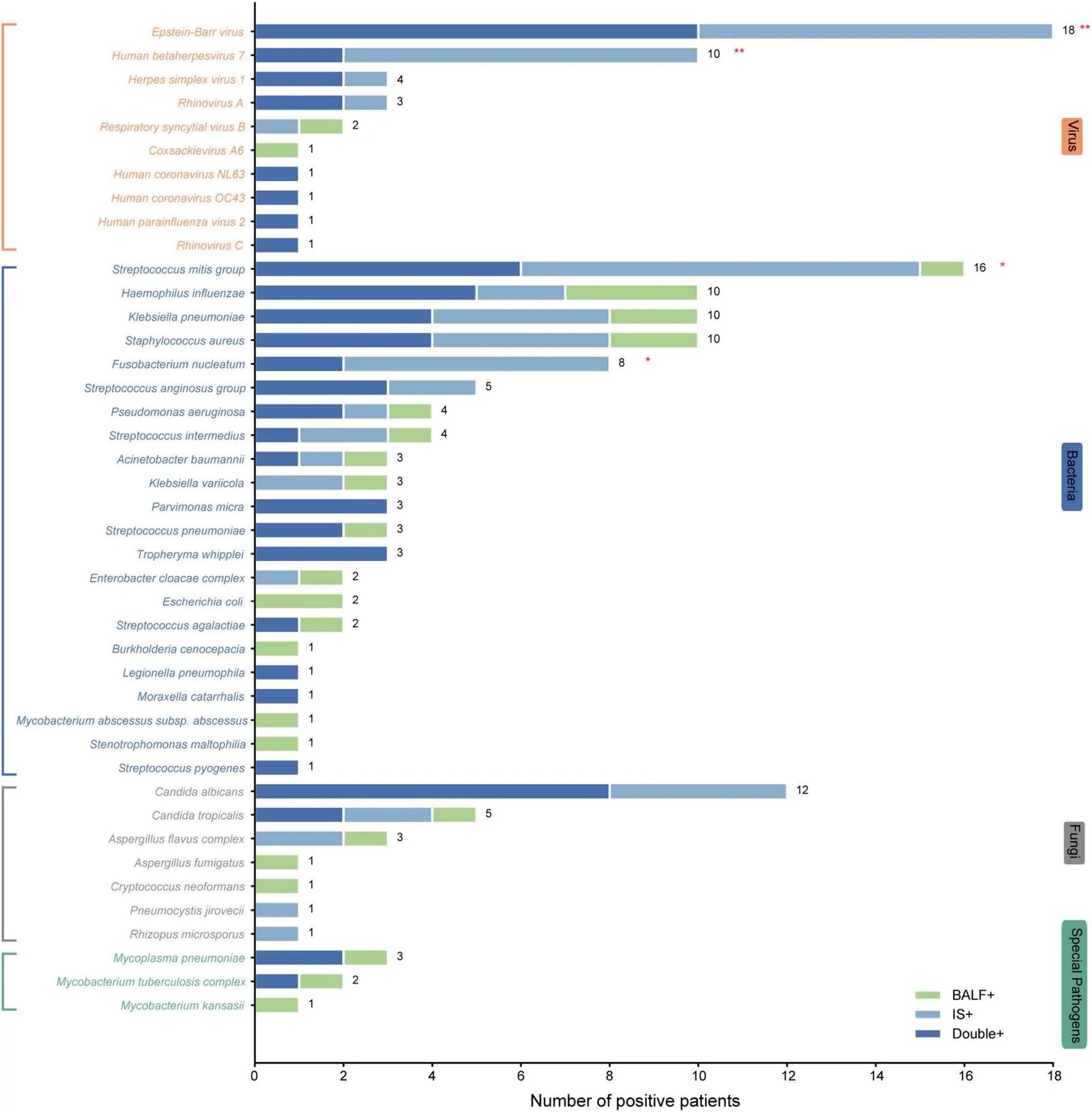
Comparison of microorganism detection results using tNGS assay in IS and BALF. Microorganisms detected by tNGS assays are categorized by biological taxonomy (viruses, bacteria, fungi, and special pathogens). Light blue bars indicate IS specimen-specific microorganisms (detected exclusively in IS). Green bars indicate BALF specimen-specific microorganisms (detected exclusively in BALF). Dark blue bars indicate pathogens detected simultaneously in both specimens. Asterisks indicate statistical significance of the detection differences between the two specimen types calculated by McNemar’s exact test: *P < 0.05; **P < 0.01.

Further, Figure 5 compares microorganism-specific paired positivity between IS and BALF by tNGS. Crucially, for the vast majority of primary respiratory pathogens, the detection performance of IS was highly concordant with BALF. For major bacterial targets (such as *Staphylococcus aureus* and *Klebsiella pneumoniae*) and dominant fungal microorganisms (such as *Candida albicans* and *Candida tropicalis*), there were no significant differences in detection rates between the two workflows. This high level of consistency confirms that IS tNGS reliably captures the core etiological profile of lower respiratory infections.

Statistically significant disparities were exclusively restricted to four specific targets: the *Streptococcus mitis* group (*P* < 0.05), *Fusobacterium nucleatum* (*P* < 0.05), *Epstein-Barr virus* (*P* < 0.01), and *Human betaherpesvirus 7* (*P* < 0.01), all of which exhibited significantly higher detection in the IS cohort. Importantly, these four microorganisms are quintessential constituents of the normal oropharyngeal flora and common salivary viral shedders. Their enriched detection in IS represents a predictable, systematic background variance intrinsic to the trans-oral expectoration route.

Taken together, the microbial spectra detected by IS and BALF workflows were highly consistent for primary lower respiratory pathogens. The statistically significant quantitative differences between the two specimens were exclusively limited to a few specific oropharyngeal opportunistic microbes, which were detected at higher frequencies in the IS cohort.

### Case-level contextual comparison with conventional microbiological tests

Conventional microbiological tests (CMTs) identified the adjudicated primary etiology in only 10.0% of cases (3/30; 95% CI 3.5–25.6%). Specifically, BALF cultures were positive in three cases, one of which also yielded a positive sputum culture. A case-level review of these three patients illustrates the diagnostic concordance and potential complementary value of tNGS alongside conventional cultures (Table 4):

- Case 1: Conventional culture isolated Staphylococcus aureus. Both IS and BALF tNGS successfully detected *S. aureus*, achieving 100% diagnostic alignment with the conventional culture result for this definitive pathogen.
- Case 2: Conventional culture isolated *Klebsiella pneumoniae*. IS tNGS successfully captured *K. pneumoniae*, fully verifying the conventional culture finding, whereas BALF tNGS failed to detect this specific target pathogen.
- Case 3: Conventional culture isolated *Klebsiella pneumoniae*. While IS tNGS confirmed the presence of *K. pneumoniae*, clinical adjudication determined the true primary driver to be the Mycobacterium tuberculosis complex (MTBC), which was exclusively captured by BALF tNGS. This indicates that the culture-isolated *K. pneumoniae* represented secondary superficial colonization rather than the primary deep-seated etiological driver.

**TABLE 4:** Horizontal comparison of identified pathogens between conventional culture and tNGS.

Case	Conventional culture (specimen)	IS tNGS finding	BALF tNGS finding
Case 1	*Staphylococcus aureus* (BALF)	*S. aureus* (+)	*S. aureus* (+)
Case 2	*Klebsiella pneumoniae* (BALF)	*K. pneumoniae* (+)	Target not detected (-)
Case 3	*Klebsiella pneumoniae* (Sputum)	*K. pneumoniae* (+)	*Mycobacterium tuberculosis complex* (+)

These case-level comparisons should be interpreted descriptively rather than as evidence that tNGS replaces conventional culture. For readily culturable bacterial pathogens such as Staphylococcus aureus and *Klebsiella pneumoniae*, culture remains clinically important because it provides viable isolates and supports antimicrobial susceptibility testing. In this cohort, tNGS provided complementary organism-level information within the composite adjudication framework, particularly when culture findings required clinical contextualization or when special pathogens were suspected.

## Discussion

Previous studies have shown that, in BALF specimens, tNGS achieves etiologic diagnostic performance comparable to mNGS (7, 15). Spontaneously expectorated sputum is highly susceptible to contamination by oropharyngeal colonizing flora (16). Accordingly, BALF is often prioritized as a more representative lower-airway specimen in routine etiologic workups. However, whether bronchoscopy-based BALF sampling should be routinely required remains a practical concern, as its invasiveness, resource utilization, and limited patient tolerance constrain broad implementation (17). By contrast, induced sputum (IS) is a less-invasive sampling option that may balance accessibility with the ability to capture clinically relevant lower-airway pathogen information. Therefore, direct validation of diagnostic concordance and applicability boundaries of IS-based tNGS within the same patient cohort is warranted, to determine whether IS can serve as a preferred first-line specimen in non-complex scenarios and help reduce unnecessary bronchoscopy.

To our knowledge, this is the first study to directly evaluate the diagnostic concordance of paired IS and BALF specimens using tNGS within the same LRTI cohort. Although BALF exhibited a numerical trend toward higher overall clinical-match positivity, our exploratory stratification by pathogenic potential suggests that this variance might be largely driven by the higher burden of oropharyngeal colonizers inherent to trans-oral sampling. Notably, after excluding background noise flora, the pathogen-capture frequencies between IS and BALF appeared highly comparable within this specific pilot cohort. These preliminary observations provide exploratory evidence that the overall discrepancy may reflect baseline microbial noise rather than a major deficit in IS diagnostic sensitivity. Rather than positioning IS as a direct alternative to BALF, our results merely suggest that IS tNGS might have potential utility as a supplementary, less-invasive sampling option in carefully selected clinical contexts, which requires further validation to define its exact clinical role. Importantly, this specimen-level interpretation should not be understood as replacing conventional culture. For readily culturable bacterial pathogens, conventional culture remains necessary because it provides viable isolates for antimicrobial susceptibility testing and therefore remains integral to antimicrobial stewardship. In this context, tNGS should be interpreted as complementary microbiological evidence within a composite clinical framework, rather than as a stand-alone trigger for antimicrobial escalation.

Based on this diagnostic stewardship framework (9, 18), IS tNGS may be considered as an initial less-invasive sampling option in selected non-complex LRTI scenarios. BALF tNGS should be prioritized—or promptly escalated to—under the following conditions: (i) clinical deterioration or severe disease requiring ICU-level care; (ii) immunocompromised status or high suspicion for opportunistic infection; (iii) imaging patterns raising suspicion for mycobacterial disease or other atypical etiologies; (iv) negative IS tNGS results or results that are clinically discordant despite persistently high clinical suspicion; and (v) situations in which bronchoscopy is indicated for broader diagnostic or therapeutic purposes (e.g., airway evaluation or lavage for cytology).

Although IS is more standardized than spontaneously expectorated sputum, it is still expelled through the oropharyngeal tract and is therefore inherently influenced by the oral microbiota, salivary components, and shedding of colonizing microorganisms or viruses from the upper airway (19). Previous studies have shown that the respiratory tract is not sterile and that noninvasive specimens are more likely to incorporate oropharyngeal background signals, resulting in a higher burden of colonizing organisms (20). Consistent with this, our species-level comparison demonstrated that the differences between IS and BALF were largely confined to a limited number of microorganisms that were more frequently detected in IS, including the *Streptococcus mitis* group, *Fusobacterium nucleatum*, Epstein–Barr virus (EBV), and human betaherpesvirus 7 (HHV-7). The *mitis* group streptococci are well-recognized dominant oral colonizers (21), while *F. nucleatum* is widely regarded as an anaerobic oral commensal and a periodontal-associated organism (22). EBV and HHV-7, in contrast, are herpesviruses rather than bacteria; accordingly, their detection in IS is more likely to reflect enrichment from oropharyngeal background, whereas detection in BALF—particularly in critically ill or immunocompromised patients—may be associated with lower-airway viral reactivation (23, 24). Therefore, when IS is used as a first-line specimen for tNGS, herpesvirus detection should not be prioritized as the primary explanation for lower respiratory tract infection in the absence of clinical features suggestive of viral disease. This distinction between clinically relevant pathogens and background flora reflects a broader challenge in respiratory metagenomics—namely, differentiating infection from colonization (25). In this context, the observation that Category A definitive pathogens were detected at comparable frequencies in IS and BALF argues that the core diagnostic information carried by the two specimen types is substantially convergent, while the divergence is largely confined to organisms of uncertain or low pathogenic potential.

Our sensitivity analysis further clarified the source of the between-specimen discrepancies. We prespecified “special pathogens” as *Mycoplasma* spp. and *Mycobacterium* spp. (including TB/NTM). After excluding these cases, the performance gap between IS and BALF narrowed substantially (clinical-match positivity, 75% vs. 83.3%; McNemar *p* = 0.688; PPA, 80.0%), suggesting that the incremental diagnostic advantage of BALF over IS is mainly concentrated in special-pathogen settings, particularly mycobacterial infection. Previous studies have shown that, in patients with suspected active or inactive tuberculosis who are unable to produce sputum or have smear-negative sputum, the diagnostic accuracy of three sequential induced sputum samples is comparable to that of BALF (26, 27). Zar et al. further reported that combining induced sputum with Xpert MTB/RIF Ultra or with other respiratory specimens can achieve very high diagnostic accuracy (28). A plausible explanation for this pattern is the difference in sampling frequency and in the burden of deep-airway pathogens. Mycobacteria are often located in the deeper lower respiratory tract, including within alveolar macrophages or the pulmonary interstitium (29). Therefore, a single IS specimen may capture insufficient pathogen biomass or nucleic acid, potentially falling below the detection threshold of tNGS and resulting in false-negative findings. The comparable performance reported in previous studies was likely achieved through repeated sampling, which compensated for the limited yield of a single IS specimen. Likewise, the concept of a “combination of specimens” strategy is consistent with our finding that combining IS and BALF increased overall clinical coverage to 93.3% (26–28).

Induced sputum has particular clinical value for patients with respiratory infections who are unable to produce sputum spontaneously. Prior studies support induced sputum as a feasible lower respiratory tract specimen, even in patients unable to readily expectorate sputum (30). For specific pathogens, induced sputum has also shown utility in the diagnosis of *Pneumocystis jirovecii* pneumonia, with a negative predictive value of 99% (31). In studies of coronavirus disease 2019, induced sputum has been reported to be more sensitive than throat swabs for detecting SARS-CoV-2 RNA in certain settings, and has been proposed as a supplementary tool for confirming viral negativity prior to discharge (32, 33). Taken together, these findings support induced sputum as a feasible and clinically acceptable noninvasive or minimally invasive lower respiratory tract specimen, while our study further extends this evidence by providing paired concordance data between induced sputum and BALF in the setting of tNGS.

Furthermore, our analysis highlights the value of tNGS in identifying fastidious organisms that evade conventional culture, with *Tropheryma whipplei* serving as a compelling specific scenario. While increasingly recognized via NGS as an emerging pulmonary pathogen (34), *T. whipplei* frequently resides in the oral cavity as a commensal, making rigorous clinical adjudication essential to differentiate true infection from background carriage (35, 36). In our cohort, *T. whipplei* was adjudicated as clinically relevant in three patients. Notably, non-invasive IS tNGS demonstrated 100% paired concordance with BALF tNGS (3 vs. 3 cases) for this fastidious target. This specific scenario illustrates that IS sampling can reliably capture complex, atypical pulmonary pathogens, potentially reducing the reliance on immediate bronchoscopy.

This study has several limitations. First, it was a single-center exploratory study with a relatively small sample size, which may limit generalizability, particularly for rare pathogens and specific subgroups. Second, although recruitment was conducted concurrently across the three campuses of our Hospital and eligibility was based on broad clinical and radiologic criteria, the short enrollment period may still have introduced seasonal bias in the observed pathogen spectrum. Third, only single IS samples were evaluated; whether serial IS sampling can further narrow the detection gap between IS and BALF warrants further study. Finally, culture-based CMTs served as a descriptive real-world comparator rather than a complete conventional reference standard.

## Conclusion

In conclusion, IS and BALF showed substantial concordance in the detection of major lower respiratory tract pathogens, while most between-specimen differences were attributable to a limited number of oropharyngeal-associated bacteria and herpesvirus background signals. In selected non-complex LRTI scenarios, IS tNGS may serve as a clinically informative, less-invasive initial sampling option, whereas BALF remains a valuable complementary specimen when special pathogens, immunocompromised status, severe disease, or unresolved diagnostic uncertainty is present.

## Data Availability

The raw data supporting the conclusions of this article will be made available by the authors, without undue reservation.

## References

[1] GBD 2019 Diseases and Injuries Collaborators. Global burden of 369 diseases and injuries in 204 countries and territories, 1990-2019: a systematic analysis for the global burden of disease study 2019. *Lancet*. (2020) 396:1204–22. 10.1016/S0140-6736(20)30925-9 33069326 PMC7567026

[2] Torres A Cilloniz C Niederman MS Menéndez R Chalmers JD Wunderink RG et al. Pneumonia. *Nat Rev Dis Primers*. (2021) 7:25. 10.1038/s41572-021-00259-0 33833230

[3] Niederman MS Torres A. Respiratory infections. *Eur Respir Rev*. (2022) 31:220150. 10.1183/16000617.0150-2022 36261160 PMC9724828

[4] Gu W Miller S Chiu CY. Clinical metagenomic next-generation sequencing for pathogen detection. *Annu Rev Pathol*. (2019) 14:319–38. 10.1146/annurev-pathmechdis-012418-012751 30355154 PMC6345613

[5] Ramanan P Bryson AL Binnicker MJ Pritt BS Patel R. Syndromic panel-based testing in clinical microbiology. *Clin Microbiol Rev*. (2017) 31:e24–17. 10.1128/CMR.00024-17 29142077 PMC5740973

[6] Gaston DC Miller HB Fissel JA Jacobs E Gough E Wu J et al. Evaluation of metagenomic and targeted next-generation sequencing workflows for detection of respiratory pathogens from bronchoalveolar lavage fluid specimens. *J Clin Microbiol*. (2022) 60:e0052622. 10.1128/jcm.00526-22 35695488 PMC9297812

[7] Ding Y Jing C Wei J Wang D Li W Wang M et al. Comparison of the diagnostic capabilities of tNGS and mNGS for pathogens causing lower respiratory tract infections: a prospective observational study. *Front Cell Infect Microbiol*. (2025) 15:1578939. 10.3389/fcimb.2025.1578939 40557317 PMC12185458

[8] Yin Y Zhu P Guo Y Li Y Chen H Liu J et al. Enhancing lower respiratory tract infection diagnosis: implementation and clinical assessment of multiplex PCR-based and hybrid capture-based targeted next-generation sequencing. *EBioMedicine*. (2024) 107:105307. 10.1016/j.ebiom.2024.105307 39226681 PMC11403251

[9] Metlay JP Waterer GW Long AC Anzueto A Brozek J Crothers K et al. Diagnosis and treatment of adults with community-acquired pneumonia. An official clinical practice guideline of the American thoracic society and infectious diseases society of America. *Am J Respir Crit Care Med*. (2019) 200:e45–67. 10.1164/rccm.201908-1581ST 31573350 PMC6812437

[10] Tao Y Yan H Liu Y Zhang F Luo L Zhou Y et al. Diagnostic performance of metagenomic next-generation sequencing in pediatric patients: a retrospective study in a large children’s medical center. *Clin Chem*. (2022) 68:1031–41. 10.1093/clinchem/hvac067 35704075

[11] Zhang F Wang H Wang W Zhu Y Mao Y Wang T et al. Induced sputum: current progress and prospect. *Eur J Med Res*. (2025) 30:795. 10.1186/s40001-025-02974-w 40855337 PMC12376322

[12] Langelier C Kalantar KL Moazed F Wilson MR Crawford ED Deiss T et al. Integrating host response and unbiased microbe detection for lower respiratory tract infection diagnosis in critically ill adults. *Proc Natl Acad Sci USA*. (2018) 115:E12353–62. 10.1073/pnas.1809700115 30482864 PMC6310811

[13] Liang M Fan Y Zhang D Yang L Wang X Wang S et al. Metagenomic next-generation sequencing for accurate diagnosis and management of lower respiratory tract infections. *Int J Infect Dis*. (2022) 122:921–9. 10.1016/j.ijid.2022.07.060 35908723

[14] Velly L Cancella de Abreu M Boutolleau D Cherubini I Houas E Aurousseau A et al. Point-of-care multiplex molecular diagnosis coupled with procalcitonin-guided algorithm for antibiotic stewardship in lower respiratory tract infection: a randomized controlled trial. *Clin Microbiol Infect*. (2023) 29:1409–16. 10.1016/j.cmi.2023.07.031 37549731

[15] Li S Tong J Liu Y Shen W Hu P. Targeted next generation sequencing is comparable with metagenomic next generation sequencing in adults with pneumonia for pathogenic microorganism detection. *J Infect*. (2022) 85:e127–9. 10.1016/j.jinf.2022.08.022 36031154

[16] Ruan Z Shi H Chang L Zhang J Fu M Li R et al. The diagnostic efficacy of metagenomic next-generation sequencing (mNGS) in pathogen identification of pediatric pneumonia using bronchoalveolar lavage fluid (BALF): a systematic review and meta-analysis. *Microb Pathog*. (2025) 203:107492. 10.1016/j.micpath.2025.107492 40113108

[17] Du Rand IA Blaikley J Booton R Chaudhuri N Gupta V Khalid S et al. British thoracic society guideline for diagnostic flexible bronchoscopy in adults: accredited by NICE. *Thorax*. (2013) 68:i1–44. 10.1136/thoraxjnl-2013-203618 23860341

[18] Kalil AC Metersky ML Klompas M Muscedere J Sweeney DA Palmer LB et al. Management of adults with hospital-acquired and ventilator-associated pneumonia: 2016 clinical practice guidelines by the infectious diseases society of America and the American thoracic society. *Clin Infect Dis*. (2016) 63:e61–111. 10.1093/cid/ciw353 27418577 PMC4981759

[19] Dickson RP Erb-Downward JR Freeman CM McCloskey L Falkowski NR Huffnagle GB et al. Bacterial topography of the healthy human lower respiratory tract. *mBio.* (2017) 8:e2287–2216. 10.1128/mBio.02287-16 28196961 PMC5312084

[20] Claassen-Weitz S Lim KYL Mullally C Zar HJ Nicol MP. The association between bacteria colonizing the upper respiratory tract and lower respiratory tract infection in young children: a systematic review and meta-analysis. *Clin Microbiol Infect*. (2021) 27:1262–70. 10.1016/j.cmi.2021.05.034 34111578 PMC8437050

[21] Mitchell J. Streptococcus mitis: walking the line between commensalism and pathogenesis. *Mol Oral Microbiol*. (2011) 26:89–98. 10.1111/j.2041-1014.2010.00601.x 21375700

[22] Brennan CA Garrett WS. *Fusobacterium nucleatum* - symbiont, opportunist and oncobacterium. *Nat Rev Microbiol*. (2019) 17:156–66. 10.1038/s41579-018-0129-6 30546113 PMC6589823

[23] Huang L Zhang X Pang L Sheng P Wang Y Yang F et al. Viral reactivation in the lungs of patients with severe pneumonia is associated with increased mortality, a multicenter, retrospective study. *J Med Virol*. (2023) 95:e28337. 10.1002/jmv.28337 36418241 PMC10099828

[24] Xu J Zhong L Shao H Wang Q Dai M Shen P et al. Incidence and clinical features of HHV-7 detection in lower respiratory tract in patients with severe pneumonia: a multicenter, retrospective study. *Crit Care*. (2023) 27:248. 10.1186/s13054-023-04530-6 37353839 PMC10290302

[25] Man WH de Steenhuijsen Piters WA Bogaert D. The microbiota of the respiratory tract: gatekeeper to respiratory health. *Nat Rev Microbiol*. (2017) 15:259–70. 10.1038/nrmicro.2017.14 28316330 PMC7097736

[26] Brown M Varia H Bassett P Davidson RN Wall R Pasvol G. Prospective study of sputum induction, gastric washing, and bronchoalveolar lavage for the diagnosis of pulmonary tuberculosis in patients who are unable to expectorate. *Clin Infect Dis*. (2007) 44:1415–20. 10.1086/516782 17479935

[27] McWilliams T Wells AU Harrison AC Lindstrom S Cameron RJ Foskin E. Induced sputum and bronchoscopy in the diagnosis of pulmonary tuberculosis. *Thorax*. (2002) 57:1010–4. 10.1136/thorax.57.12.1010 12454293 PMC1758793

[28] Zar HJ Workman LJ Prins M Bateman LJ Mbhele SP Whitman CB et al. Tuberculosis diagnosis in children using xpert ultra on different respiratory specimens. *Am J Respir Crit Care Med*. (2019) 200:1531–8. 10.1164/rccm.201904-0772OC 31381861 PMC6909828

[29] Cohen SB Gern BH Delahaye JL Adams KN Plumlee CR Winkler JK et al. Alveolar macrophages provide an early mycobacterium tuberculosis niche and initiate dissemination. *Cell Host Microbe.* (2018) 24:439e–46e. 10.1016/j.chom.2018.08.001 30146391 PMC6152889

[30] Lahti E Peltola V Waris M Virkki R Rantakokko-Jalava K Jalava J et al. Induced sputum in the diagnosis of childhood community-acquired pneumonia. *Thorax*. (2009) 64:252–7. 10.1136/thx.2008.099051 19052043

[31] Turner D Schwarz Y Yust I. Induced sputum for diagnosing Pneumocystis carinii pneumonia in HIV patients: new data, new issues. *Eur Respir J*. (2003) 21:204–8. 10.1183/09031936.03.00035303 12611375

[32] Lai T Xiang F Zeng J Huang Y Jia L Chen H et al. Reliability of induced sputum test is greater than that of throat swab test for detecting SARS-CoV-2 in patients with COVID-19: a multi-center cross-sectional study. *Virulence*. (2020) 11:1394–401. 10.1080/21505594.2020.1831342 33073676 PMC7575004

[33] Han H Luo Q Mo F Long L Zheng WSARS-. CoV-2 RNA more readily detected in induced sputum than in throat swabs of convalescent COVID-19 patients. *Lancet Infect Dis*. (2020) 20:655–6. 10.1016/S1473-3099(20)30174-2 32171389 PMC7271222

[34] Tao Y Du R Mao H. Tropheryma whipplei pneumonia revealed by Metagenomic next-generation sequencing: report of two cases. *Diagn Microbiol Infect Dis*. (2024) 110:116427. 10.1016/j.diagmicrobio.2024.116427 39024936

[35] Lai LM Zhu XY Zhao R Chen Q Liu JJ Liu Y et al. Tropheryma whipplei detected by metagenomic next-generation sequencing in bronchoalveolar lavage fluid. *Diagn Microbiol Infect Dis*. (2024) 109:116374. 10.1016/j.diagmicrobio.2024.116374 38805857

[36] Sun L Wang Y Fang J Li Z Yin Y Guo Y et al. Clinical experience with metagenomic next-generation sequencing (mNGS) for the detection of Tropheryma whipplei in respiratory specimens: a multicenter retrospective observational study. *Int J Infect Dis*. (2026) 165:108457. 10.1016/j.ijid.2026.108457 41654251

