# Supplementary material for: Induced sputum versus bronchoalveolar lavage in lower respiratory tract infection: an exploratory paired diagnostic study
Source: Front Med (Lausanne). 2026 Aug 31;13:1845078. doi: 10.3389/fmed.2026.1845078 (PMC13572613; doi:10.3389/fmed.2026.1845078)
Supplement: Supplementary file 1 [file Table_1.docx]

| **Supplementary Table 1 The standards for final clinical diagnosis** | |
| --- | --- |
| **Clinically concordant** | - If a microorganism was detected in both paired IS and BALF tNGS and was consistent with the patient’s clinical presentation and imaging findings, the result was considered clinically concordant. |
|  | - If a microorganism detected by either IS or BALF tNGS was also supported by conventional microbiological testing and received clinical agreement, then the result was considered clinically concordant. |
|  | - If a microorganism was detected only by tNGS while conventional microbiological tests were negative, but the result was compatible with the radiographic pattern, host factors, and treatment response, then the result was considered clinically concordant. |
|  | - If both tNGS and conventional microbiological tests were negative, and the final adjudication did not support an alternative microbiologically proven pathogen, then the negative result was considered clinically concordant. |
| **clinical ambiguity** | - The results of tNGS can be either negative or positive despite negative findings from conventional culture. However, the clinical significance of these results remains unclear. |
|  | - If a background-prone organism (e.g., oral commensals, Candida spp. in respiratory samples, or herpesviruses in induced sputum) was detected without clear supporting clinical evidence, then the result was considered clinically ambiguous. |
|  | - If multiple microorganisms were detected but no single organism could be assigned as the primary etiology after integrating clinical, radiologic, and microbiological findings, then the result was considered clinically ambiguous. |
| **clinical disagreement** | - If a detected microorganism was inconsistent with the patient’s syndrome, imaging pattern, host status, and treatment response, then the result was considered clinically discordant. |
|  | - If an alternative pathogen or a noninfectious diagnosis better explained the clinical presentation, then the result was considered clinically discordant. |
|  | - If both tNGS and conventional microbiological tests were negative, but the clinical course strongly supported an infectious etiology not captured by the available tests, then the result was considered clinically discordant. |
| **Definition used in the main analysis** | - Only microorganisms classified as clinically concordant with the adjudicated primary etiology were counted as clinical-match positive in the main analysis. |
|  | - Microorganisms classified as clinically ambiguous or clinically discordant were retained in descriptive spectrum analyses but were not counted as etiologic matches. |
